# Supplementary material for: Comparative Analysis of Radical Adduct Formation (RAF) Products and Antioxidant Pathways between Myricetin-3-O-Galactoside and Myricetin Aglycone
Source: Molecules. 2019 Jul 30;24(15):2769. doi: 10.3390/molecules24152769 (PMC6696482; doi:10.3390/molecules24152769)
Supplement: Supplementary file 1 [file molecules-24-02769-s001.zip › Suppl. 1 flavonol 3-O-galactoside and its aglycone.pdf]

Suppl. 1 flavonol-3-*O*-galactoside and its aglycone

Comparative Analysis of Radical Adduct Formation (RAF) Products and Antioxidant Pathways Between Myricetin-3-*O*-Galactoside and Myricetin Aglycone

Xican Li [1,2,†,*], Xiaojian Ouyang [1,2,†], Minshi Liang [1,2] and Dongfeng Chen [3,4,*]

1 Innovative Research & Development Laboratory of TCM of Guangdong Province, University of Chinese Medicine, Guangzhou 510006, China
2 School of Chinese Herbal Medicine; Guangzhou University of Chinese Medicine, Guangzhou 510006, China
3 School of Basic Medical Science, Guangzhou University of Chinese Medicine, Guangzhou 510006, China
4 The Research Center of Integrative Medicine, Guangzhou University of Chinese Medicine, Guangzhou 510006, China
* Correspondence: lixican@126.com (X.L.); chen888@gzucm.edu.cn (D.C.)
† These authors contributed equally to this work.

**flavonol 3-*O*-galactoside**	**flavonol aglycone**	**plants**	**references**
isorhamnetin 3-*O*-galactoside 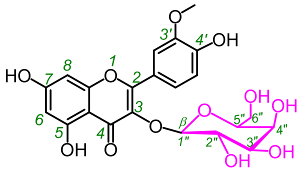	isorhamnetin 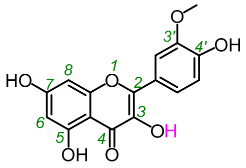	Cryptocarya alba	[7]
quercetin-3-*O*-galactoside (hyperin) 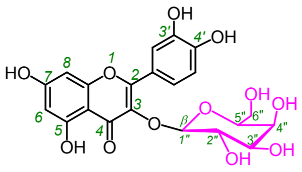	quercetin 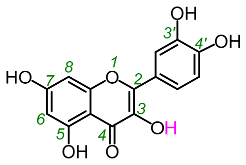	Cryptocarya alba and Cratoxylum formosum	[7], [8]
kaempferol-3-*O*-galactoside (trifolin) 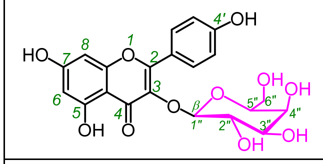	kaempferol 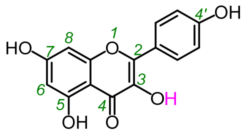	Cryptocarya alba and Consolida oliveriana	[7], [9]
syringetin-3-*O*-galactoside	syringetin	grape	[10]

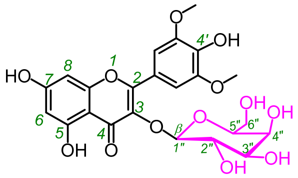	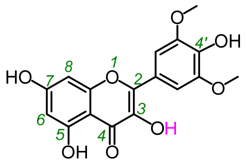		
laricitrin-3-*O*-galactoside 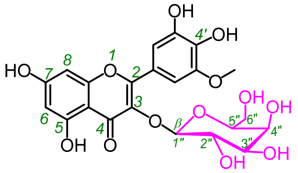	laricitrin 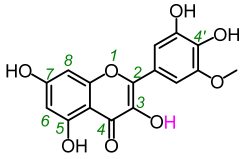	grape	[10]
myricetin-3-*O*-galactoside 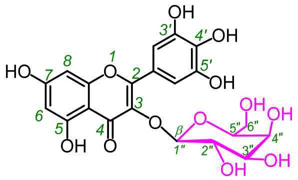	myricetin 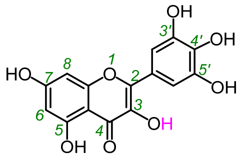	white myrtle and Nelumbo nucifera	[11], [12]
rhamnetin-3-*O*-galactoside 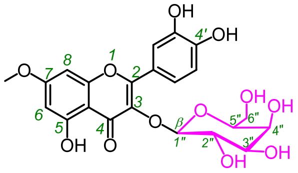	rhamnetin 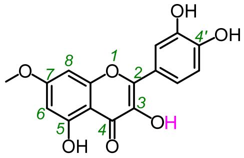		
eupalitin 3-*O*-galactoside 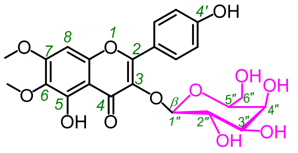	eupalitin 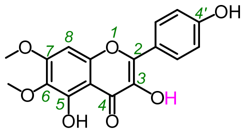		
6-methoxykaempferol 3-*O*-galactoside 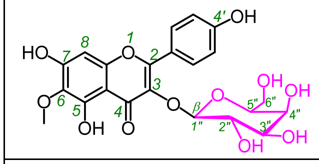	6-methoxykaempferol 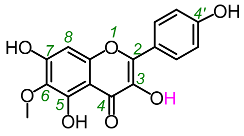		
6-methoxyquercetin 3-*O*-galactoside	6-methoxyquercetin		

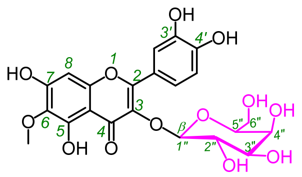	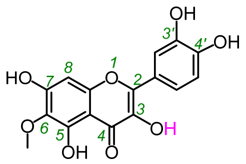		

References

7. Timmermann, B.N.; Valcic, S.; Liu, Y.L.; Montenegro, G. Flavonols from Cryptocarya alba. *Z. Naturforsch. C.* **1995**, *50*, 898-899.
8. Choi, S.J.; Tai, B.H.; Cuong, N.M.; Kim, Y.H.; Jang, H.D. Antioxidative and anti-inflammatory effect of quercetin and its glycosides isolated from mampat (Cratoxylum formosum). *Food Sci. Biotechnol.* **2012**, *21*, 587-595.
9. Diaz, J.G.; Carmona, A.J.; Torres, F.; Quintana, J.; Estevez, F.; Herz, W. Cytotoxic activities of flavonoid glycoside acetates from Consolida oliveriana. *Planta Med.* **2008**, *74*, 171-174.
10. Mattivi, F.; Guzzon, R.; Vrhovsek, U.; Stefanini, M.; Velasco, R. Metabolite profiling of grape: Flavonols and anthocyanins. *J. Agric. Food Chem.* **2006**, *54*, 7692-7702.
11. Serreli, G.; Jerkovic, I.; Gil, K.A.; Marijanovic, Z.; Pacini, V.; Tuberoso, C.I.G. Phenolic Compounds, Volatiles and Antioxidant Capacity of White Myrtle Berry Liqueurs. *Plant Food Hum. Nutr.* **2017**, *72*, 205-210.
12. Chen, S.; Fang, L.C.; Xi, H.F.; Guan, L.; Fang, J.B.; Liu, Y.L.; Wu, B.H.; Li, S.H. Simultaneous qualitative assessment and quantitative analysis of flavonoids in various tissues of lotus (Nelumbo nucifera) using high performance liquid chromatography coupled with triple quad mass spectrometry. *Anal. Chim. Acta* **2012**, *724*, 127-135.
