# Supplementary material for: Comparative Analysis of Radical Adduct Formation (RAF) Products and Antioxidant Pathways between Myricetin-3-O-Galactoside and Myricetin Aglycone
Source: Molecules. 2019 Jul 30;24(15):2769. doi: 10.3390/molecules24152769 (PMC6696482; doi:10.3390/molecules24152769)

Suppl. 2 The mass spectrum of DPPH free radical detected in negative ion mode

Comparative Analysis of Radical Adduct Formation (RAF) Products and Antioxidant Pathways Between Myricetin-3-*O*-Galactoside and Myricetin Aglycone

Xican Li [1,2,†,*], Xiaojian Ouyang [1,2,†], Minshi Liang [1,2] and Dongfeng Chen [3,4,*]

1 Innovative Research & Development Laboratory of TCM of Guangdong Province, University of Chinese Medicine, Guangzhou 510006, China
2 School of Chinese Herbal Medicine; Guangzhou University of Chinese Medicine, Guangzhou 510006, China
3 School of Basic Medical Science, Guangzhou University of Chinese Medicine, Guangzhou 510006, China
4 The Research Center of Integrative Medicine, Guangzhou University of Chinese Medicine, Guangzhou 510006, China
* Correspondence: lixican@126.com (X.L.); chen888@gzucm.edu.cn (D.C.)
† These authors contributed equally to this work.

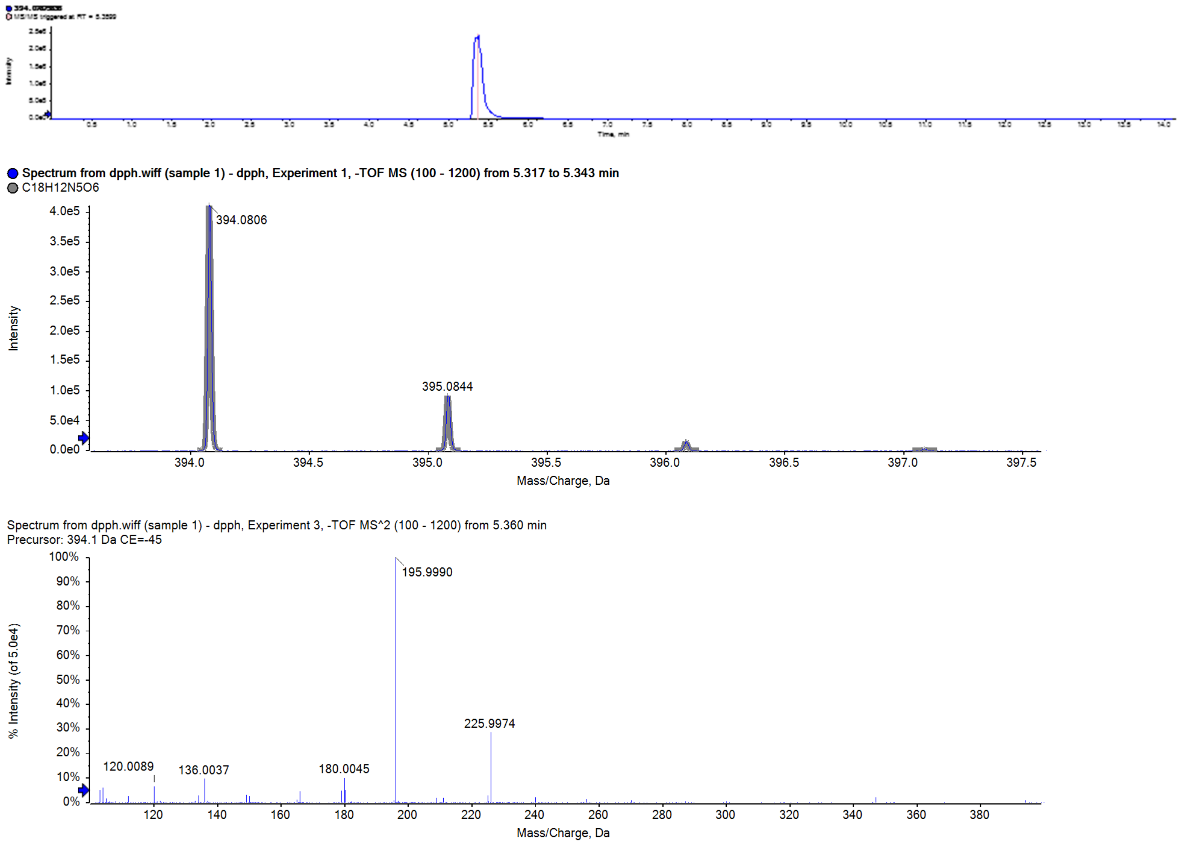

Supplement: Supplementary file 1 [file molecules-24-02769-s001.zip › Suppl. 2 The mass spectrum of DPPH free radicals detected in negative ion mode.pdf]
