# Supplementary material for: Comparative Analysis of Radical Adduct Formation (RAF) Products and Antioxidant Pathways between Myricetin-3-O-Galactoside and Myricetin Aglycone
Source: Molecules. 2019 Jul 30;24(15):2769. doi: 10.3390/molecules24152769 (PMC6696482; doi:10.3390/molecules24152769)
Supplement: Supplementary file 1 [file molecules-24-02769-s001.zip › Suppl. 3 Dose response curves Figure S1-S4.pdf]

Suppl. 3 Dose response curves Figure S1-S4

Comparative Analysis of Radical Adduct Formation (RAF) Products and Antioxidant Pathways Between Myricetin-3-*O*-Galactoside and Myricetin Aglycone

Xican Li [1,2,†,*], Xiaojian Ouyang [1,2,†], Minshi Liang [1,2] and Dongfeng Chen [3,4,*]

[1] Innovative Research & Development Laboratory of TCM of Guangdong Province, University of Chinese Medicine, Guangzhou 510006, China
[2] School of Chinese Herbal Medicine; Guangzhou University of Chinese Medicine, Guangzhou 510006, China
[3] School of Basic Medical Science, Guangzhou University of Chinese Medicine, Guangzhou 510006, China
[4] The Research Center of Integrative Medicine, Guangzhou University of Chinese Medicine, Guangzhou 510006, China
* Correspondence: lixican@126.com (X.L.); chen888@gzucm.edu.cn (D.C.)
† These authors contributed equally to this work.

Note: This Supporting information provides the original data of **Table 1** in the main text. All data underline are mentioned in **Table 1** in the main text.

1. DPpH•-trapping assay

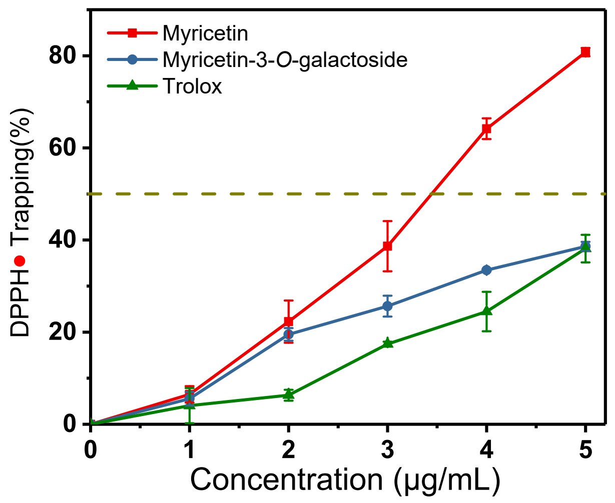

Figure S1: The dose response curves of myricetin, myricetin-3-*O*-galactoside in DPpH•-trapping assay. Each value is expressed as mean ± SD (n = 3).

Tab. S1 The comparison of IC_{50} values of myricetin, myricetin-3-*O*-galactoside and positive control in DPpH•- trapping assay.

	Mean±SD μg/mL	Mean±SD μM
Myricetin	3.4 ± 0.1	10.7 ± 0.3 [a]
Myricetin-3-*O*-galactoside	6.2 ± 0.1	12.9 ± 0.3 [b]
Trolox	6.6 ± 0.6	26.4 ± 2.5

IC_{50} value was defined as the concentration of 50% superoxide anion radical inhibition and calculated by linear regression which was analyzed by Origin 6.0 professional software. Means values with different superscripts in the same column are significantly different (p<0.05).

2. PTIO•- trapping assay(pH 4.5)

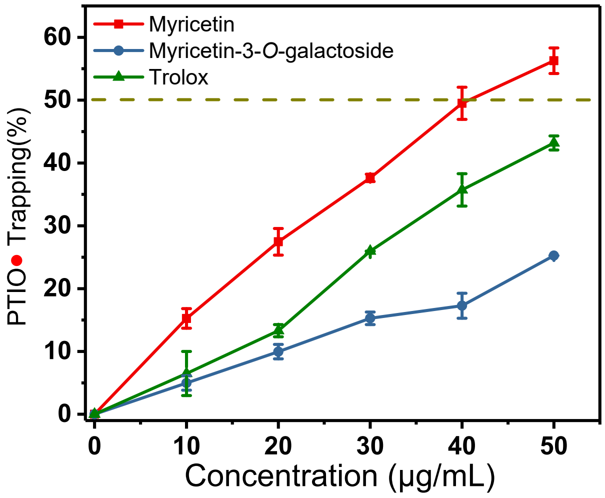

Figure S2**:** The dose response curves of myricetin, myricetin-3-*O*-galactoside and Trolox in PTIO•-trapping assay (pH 4.5). The value is expressed as mean ± SD (n = 3).

Tab. S2 The comparison of IC_{50} values of myricetin, myricetin-3-*O*-galactoside and positive control in PTIO•-trapping assay.

	Mean±SD μg/mL	Mean±SD μM
myricetin	42.3± 1.6	132.9 ± 5.1 [a]
Myricetin-3-*O*-galactoside	126.7 ±1.7	263.7 ± 3.5 [b]
Trolox	55.1 ± 1.1	220.1 ± 4.6

IC_{50} value was defined as the concentration of 50% superoxide anion radical inhibition and calculated by linear regression which was analyzed by Origin 6.0 professional software. Means values with different superscripts in the same column are significantly different (p<0.05).

3. PTIO•-trapping assay(pH 7.4)

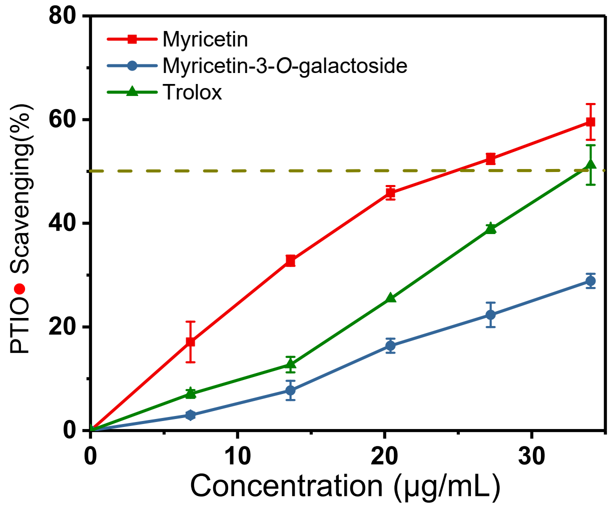

Figure S3**:** The dose response curves of myricetin, myricetin-3-*O*-galactoside and Trolox in PTIO•-trapping assay (pH 7.4). The value is expressed as mean ± SD (n = 3).

Tab. S3 The comparison of IC_{50} values of myricetin, myricetin-3-*O*-galactoside and positive control in PTIO•-trapping assay.

	Mean±SD μg/mL	Mean±SD μM
Myricetin	25.9± 0.8	81.5 ± 2.4 [a]
Myricetin-3-*O*-galactoside	63.0 ± 2.5	131.2 ± 5.1 [b]
Trolox	35.7 ± 1.2	142.9 ± 5.0

IC_{50} value was defined as the concentration of 50% superoxide anion radical inhibition and calculated by linear regression which was analyzed by Origin 6.0 professional software. Means values with different superscripts in the same column are significantly different (p<0.05).

4. Superoxide anion (•O_2^-) scavenging assay

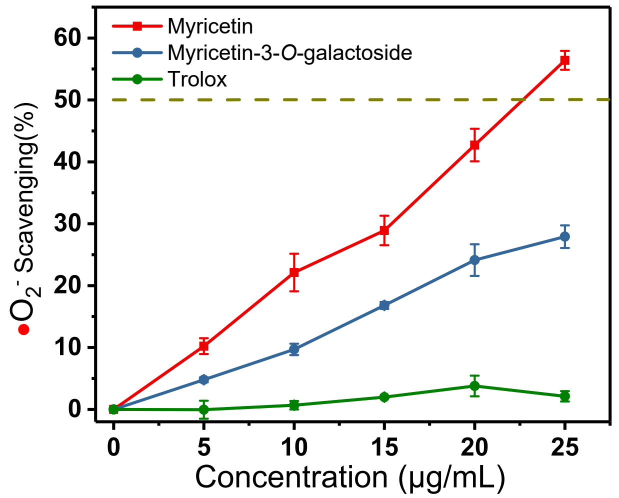

Figure S4: The dose response curves of myricetin, myricetin-3-*O*-galactoside in Superoxide anion (•O_2^-) scavenging assay. Each value is expressed as mean ± SD (n = 3).

Tab. S4 The comparison of IC_{50} values of myricetin, myricetin-3-*O*-galactoside and positive control in Superoxide anion (•O_2^-) scavenging assay.

	Mean±SD μg/mL	Mean±SD μM
Myricetin	23.3 ± 0.6	73.3 ± 1.9 [a]
Myricetin-3-*O*-galactoside	42.7 ± 3.5	88.9 ± 7.2 [b]
Trolox	695.5 ± 8.8	2777.5 ± 35.3

IC_{50} value was defined as the concentration of 50% superoxide anion radical inhibition and calculated by linear regression which was analyzed by Origin 6.0 professional software. Means values with different superscripts in the same column are significantly different ($p<0.05$).
