# Supplementary material for: Comparative Analysis of Radical Adduct Formation (RAF) Products and Antioxidant Pathways between Myricetin-3-O-Galactoside and Myricetin Aglycone
Source: Molecules. 2019 Jul 30;24(15):2769. doi: 10.3390/molecules24152769 (PMC6696482; doi:10.3390/molecules24152769)
Supplement: Supplementary file 1 [file molecules-24-02769-s001.zip › Suppl. 4 Certificate of analysis of Myricetin-3-O-galactoside.pdf]

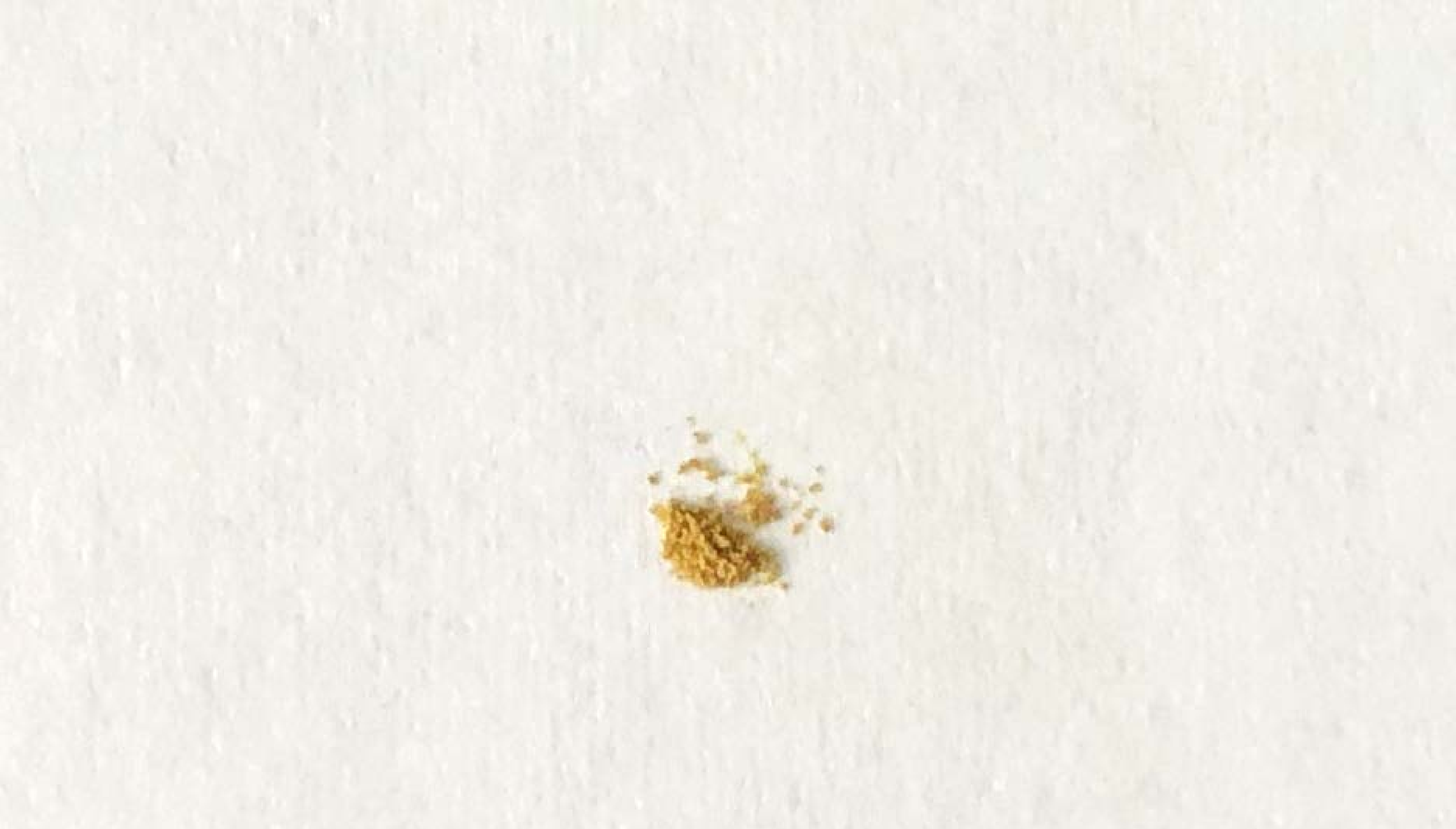

Myricetin-3-*O*-galactoside CAS: 15648-86-9

西力生物
BBP BioBioPha

Natural Product Library for Drug Discovery

CERTIFICATE OF ANALYSIS

BBP No.: BBP02385

CAS No.: 15648-86-9

Chemical Name: Myricetin 3-O-galactoside

Molecular Formula: $C_{21}H_{20}O_{13}$

Structure:

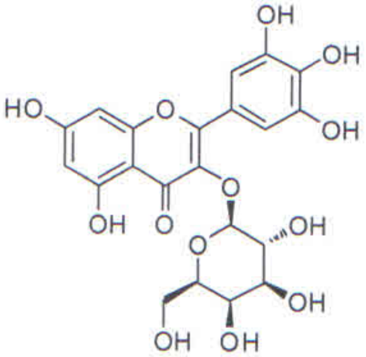

Purity: 98%

Appearance: Yellow powder

Solvent: Dimethyl sulfoxide, methanol

Exact Weight: 5.1 mg

Storage: Store in a dark place under the temperature of 0–4 °C

Intended Use: For laboratory use only

Reference: L. Y. Foo, Phytochemistry, 2000, 54(5), 539-548

Warm Notice: When publishing, please cite as: ***chemical name*** was purchased from BioBioPha Co., Ltd. (Kunming, China)

Characterization Data Summary

Analytical Test	Results
Identification by ^{1}H-NMR	Consistent with the above structure
Purity tested by HPLC, ^{1}H-NMR	98%

Authorized Signature:

Date: 2019.02.19

PASS

Tel.: +86 871 6521 7109 Fax: +86 871 6521 5563 E-mail: sales@mail.biobiopha.com

西力生物
BBP BioBioPha

Natural Product Library for Drug Discovery

PRODUCT QUALITY REPORT

Product Number: BBP02385

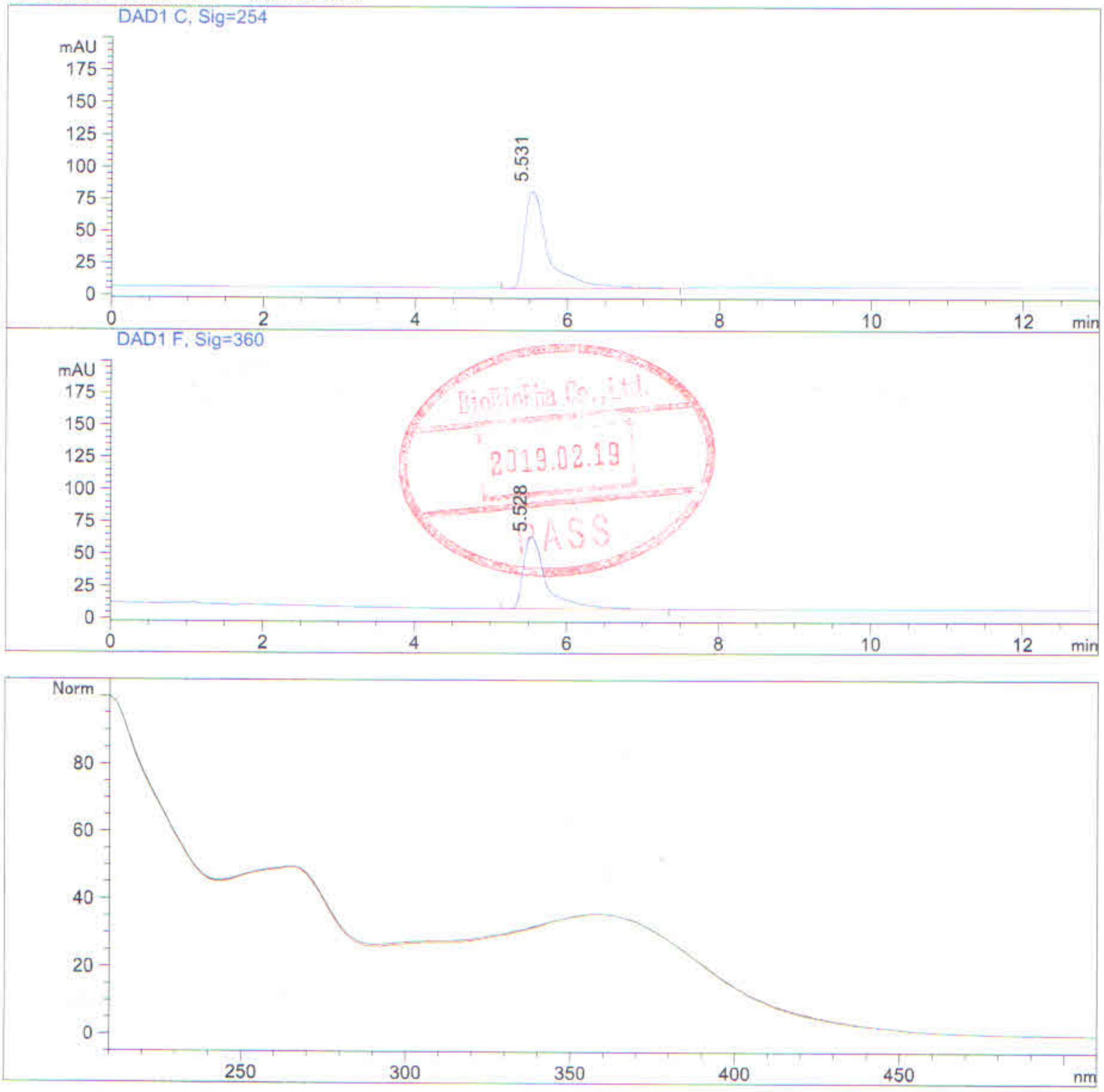

Agilent 1200 series HPLC system
Thermo Hypersil BDS C18 column (5 μm, 4.6 × 150 mm)
20%→100% MeOH in H_2O over 8.0 min followed by 100% MeOH to 13.0 min .
1.0 ml/min, 20°C

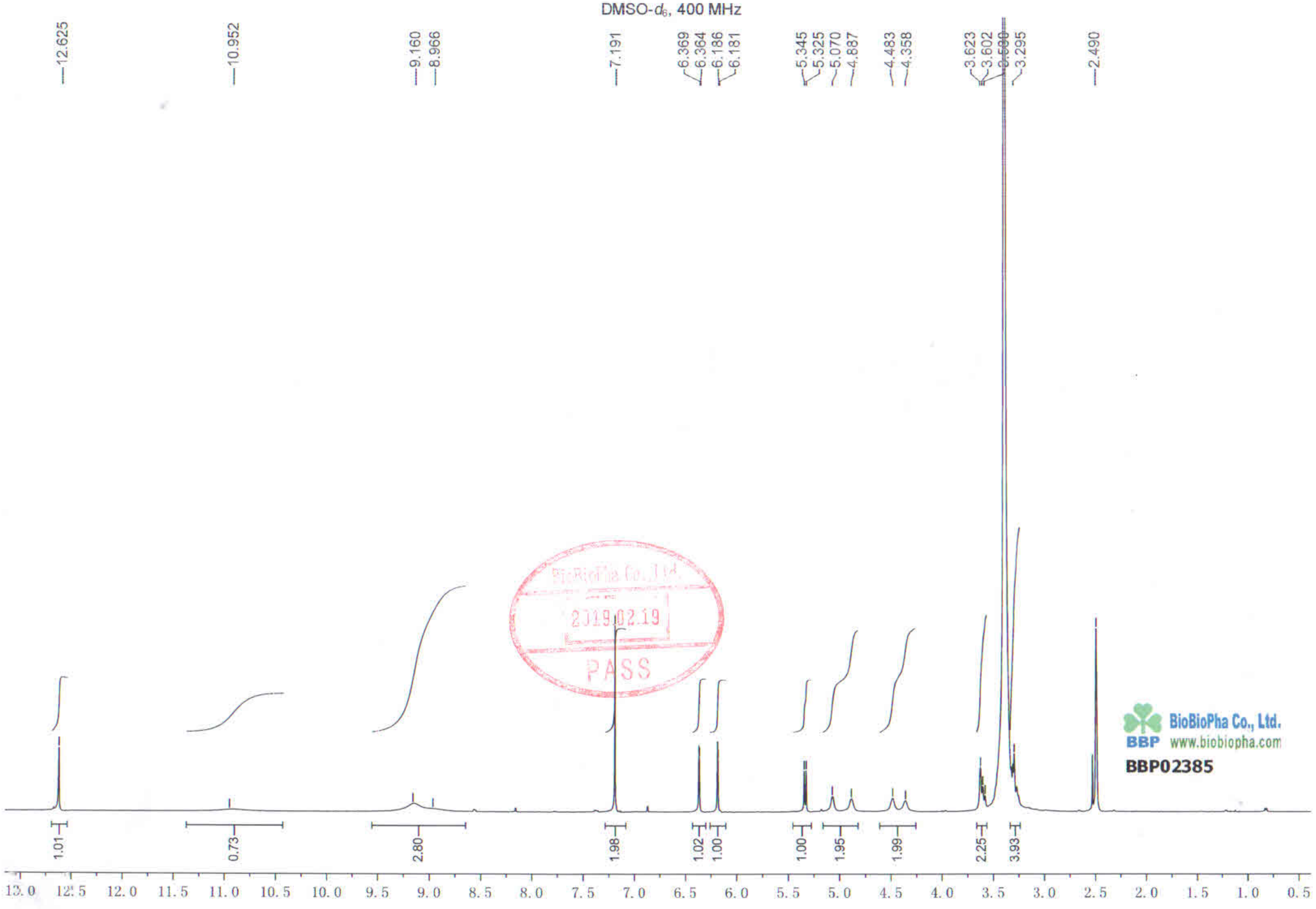

BioBioPha Co., Ltd.
BBP www.biobiopha.com
BBP02385
