# Supplementary material for: Comparative Analysis of Radical Adduct Formation (RAF) Products and Antioxidant Pathways between Myricetin-3-O-Galactoside and Myricetin Aglycone
Source: Molecules. 2019 Jul 30;24(15):2769. doi: 10.3390/molecules24152769 (PMC6696482; doi:10.3390/molecules24152769)
Supplement: Supplementary file 1 [file molecules-24-02769-s001.zip › Suppl. 5 Certificate of analysis of Myricetin.pdf]

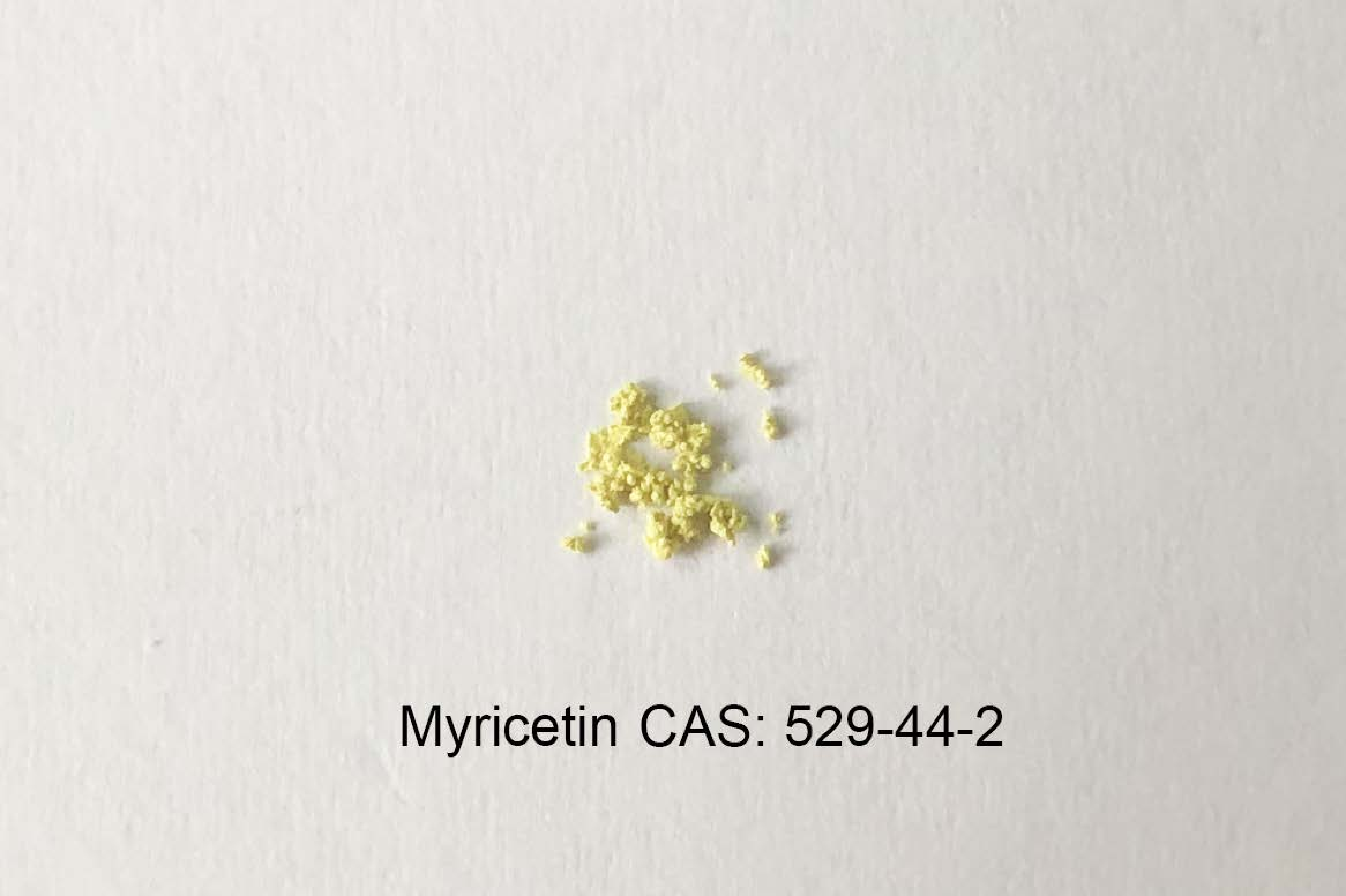
Myricetin CAS: 529-44-2

产品分析证书
Certificate of Analysis

中文名称：杨梅素

English Name：Myricetin
别名(Alias): Cannabiscetin; Myricetol

产品编码(Cat. No.):BP0970
CAS Number: 529-44-2
分子式(M. F.): C15H10O8
分子量(M. W.): 318.237

批号(Batch No.): PRF8082121
报告日期(Report date): 2017-08-21

Structure

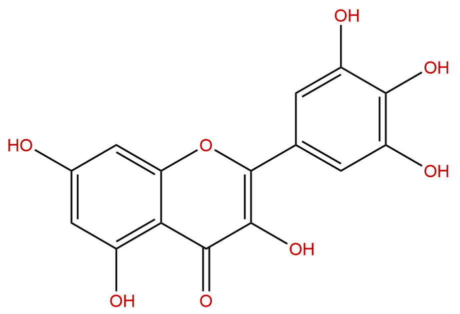

检验结果（Analytical result）:

检验项目（Test Item）	检验指标（Specifications）	检验结果（Results）
外观Appearance	Off-white powder	Light yellow powder
干燥失重Loss on drying	＜3.0%	1.4%
纯度Purity (HPLC-DAD,373nm)*	≥98.0%	99.72%
质谱Mass	318.2±1	Conforms
核磁NMR	Comply with the structure	Conforms
含量Assay	≥98.0%	103.9%

*** 色谱图见附件（Please find HPLC chromatography attached.）**

检测方法（Test Method）：Column: Agilent 5TC-C18，4.6*250mm; **Column temperature**: 30℃; **Detection Mode:** UV373nm;**Flow Rate:** 1.0ml/min; **Sample dissolution:** Methanol; **Mobile Phase: A,** 0.1% phosphoric acid in water **B,** Acetonitrile;**Gradient elution:** B, 25%-35%,15min.

贮存条件（Storage）:2~8℃, protected from light, keep package airproofed when not in use.

复测期（Retest date）:two years (2019-08-20) under conditions list above.

QC: Zhang Ling

Date: 2017-08-21

QA: Wu Qi

Date: 2017-08-21

备注(Remarks): The sample solutions should be prepa[illegible] same day, it is the best preparing the solutions immediately before use. If the solutons have to be made up in advance, it should be made as aliquots in tightly sealed vials at less than -20℃. Generally, these might be useable for up to two weeks.

In case of quality issue, please contact us within 15 days after receipt of the product.

成都普瑞法科技开发有限公司 质检专用章 Chengdu Biopurify Phytochemicals Ltd. Quality Approval Seal

Tel：+86-28-82633397 Fax：+86-28-82633165
http://www.phytopurify.com Email：sales@biopurify.com biopurify@gmail.com

Chengdu Biopurify Phytochemicals Ltd.

SAMPLE INFORMATION

Sample Name:	Myricetin PRF8082121	Acquired By:	System
Sample Type:	Unknown	Sample Set Name:	
Vial:	51	Acq. Method Set:	Myricetin
Injection #:	1	Processing Method:	Samples
Injection Volume:	10.00 ul	Channel Name:	373.0nm
Run Time:	25.0 Minutes	Proc. Chnl. Descr.:	PDA 373.0 nm
Date Acquired:	2017-8-21 12:28:29 CST		
Date Processed:	2017-8-21 13:59:36 CST		

Auto-Scaled Chromatogram

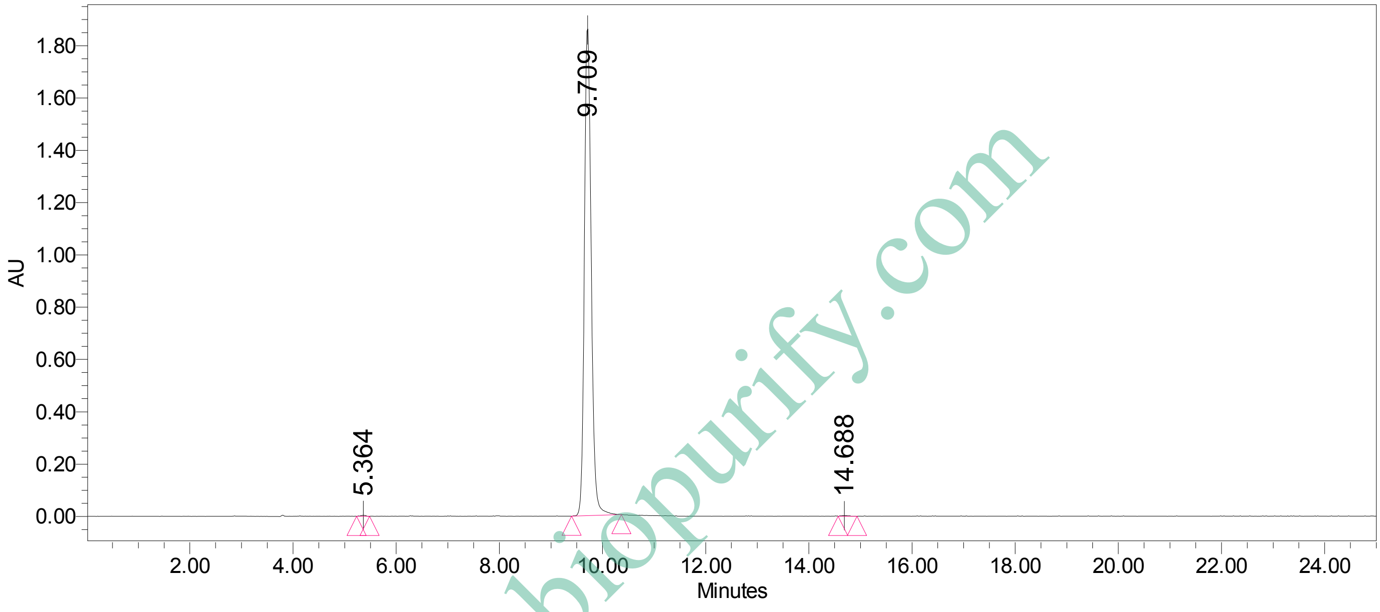

www.biopurify.com

Peak Results

	Name	RT	Area	% Area	USP Plate Count	USP Resolution
1		5.364	25973	0.15	14501.5	
2		9.709	17270921	99.72	26932.8	20.8
3		14.688	22926	0.13	22967.8	19.6
